# The global distribution of the macrolide esterase EstX from the alpha/beta hydrolase superfamily

**DOI:** 10.1038/s42003-024-06473-2

**Published:** 2024-06-28

**Authors:** Jiafu Lin, Hua Lv, Tiantian Wang, Hongkun Tao, Yi Zhong, Yang Zhou, Yibo Tang, Feng Xie, Guoqing Zhuang, Changwen Xu, Yiwen Chu, Xinrong Wang, Yongqiang Yang, Tao Song

**Affiliations:** 1 Antibiotics Research and Re-evaluation Key Laboratory of Sichuan Province, Sichuan Industrial Institute of Antibiotics, School of pharmacy, Chengdu University, 610106 Chengdu, China; 2 Sichuan Academy of Forestry, 610081 Chengdu, Sichuan China; 3 Center of Infectious Diseases, Center for Pathogen Research, West China Hospital, Sichuan University, Chengdu, China

**Keywords:** Molecular biology, Microbiology, Biochemistry

## Abstract

Macrolide antibiotics, pivotal in clinical therapeutics, are confronting resistance challenges mediated by enzymes like macrolide esterases, which are classified into Ere-type and the less studied Est-type. In this study, we provide the biochemical confirmation of EstX, an Est-type macrolide esterase that initially identified as unknown protein in the 1980s. EstX is capable of hydrolyzing four 16-membered ring macrolides, encompassing both veterinary (tylosin, tidipirosin, and tilmicosin) and human-use (leucomycin A5) antibiotics. It uses typical catalytic triad (Asp233-His261-Ser102) from alpha/beta hydrolase superfamily for ester bond hydrolysis. Further genomic context analysis suggests that the dissemination of *estX* is likely facilitated by mobile genetic elements such as integrons and transposons. The global distribution study indicates that bacteria harboring the *estX* gene, predominantly pathogenic species like *Escherichia coli*, *Salmonella enterica*, and *Klebsiella pneumoniae*, are prevalent in 74 countries across 6 continents. Additionally, the emergence timeline of the *estX* gene suggests its proliferation may be linked to the overuse of macrolide antibiotics. The widespread prevalence and dissemination of Est-type macrolide esterase highlight an urgent need for enhanced monitoring and in-depth research, underlining its significance as an escalating public health issue.

## Abstract (web summary)

Macrolide esterase EstX can degrade both veterinary and human-use 16-membered ring macrolides. This enzyme is widely distributed among pathogenic species and across 74 countries on 6 continents,with its dissemination likely facilitated by integrons and transposons.

## Introduction

Macrolide antibiotics, one of the earliest classes of antibiotics clinically employed since 1952, currently rank third in global antibiotic sales with a market value of $4.8 billion^1^. They have a characteristic macrolide ring structure and inhibit protein synthesis by binding to the 50S ribosomal subunit, thereby inhibiting bacterial growth^2–4^. Macrolide antibiotics are classified based on their macrolide ring size into three types: 14-membered ring (erythromycin, clarithromycin, roxithromycin, telithromycin), 15-membered ring (azithromycin and tulathromycin), and 16-membered ring (tylosin, tilmicosin, josamycin, tildipirosin, and spiramycin)^2,5–7^. Macrolide antibiotics are particularly effective against Gram-positive bacteria and also demonstrate efficacy against several Gram-negative pathogens in the upper respiratory tract. Commonly used macrolide antibiotics, such as azithromycin, clarithromycin, and erythromycin, are employed to treat various infections, including pneumonia, sinusitis, pharyngitis, tonsillitis, and respiratory tract infections in animals^8^. However, the long-term and extensive use of macrolide antibiotics has contributed to the increased dissemination of antimicrobial resistance. According to the PROTEKT study, 36.8% (7420/20142) of *Streptococcus pneumoniae* clinical strains isolated from 40 countries between 2001 and 2004 demonstrated resistance to macrolides^9^. In China, macrolide resistance was observed in 68.7–100% of clinical isolates of *Mycoplasma pneumoniae*^10^.

Macrolide esterases (MEs) are enzymes that degrade the lactone rings in macrolide antibiotics, rendering them ineffective, and are key resistance genes contributing to macrolide resistance in bacteria. Currently, macrolide esterases are classified into two major classes: Ere-type and EstT-type. The Ere-type includes five members: EreA and EreB, found in clinical isolates of *Escherichia coli*; EreC in multidrug-resistant (MDR) *Klebsiella pneumoniae*; and EreD in the duck pathogen *Riemerella anatipestifer*^11^. In contrast, the recently discovered EstT-type belongs to the α/β-hydrolase family, with only one characterized member to date (EstT). EstT has been detected in MDR *Sphingobacterium faecium* from watering bowl environments, predominantly found in animal microbiomes and occasionally in human microbiomes^4^. The current understanding of the distribution and transmission of both Ere-type and EstT-type macrolide esterases is limited, with only a few identified members. This limited knowledge impedes the development of strategies to combat antibiotic resistance, posing potential risks for unexpected outbreaks of resistant bacteria and implications for public health.

The Ere-type and EstT-type macrolide esterases exhibit distinct substrate specificities. Ere-type enzymes target 14-, 15-, and 16-membered macrolides commonly used in human and veterinary medicine, such as erythromycin, clarithromycin, roxithromycin, azithromycin, and josamycin^2,10,12^. In contrast, the EstT-type (EstT) specifically degrades 16-membered macrolides, like tylosin, tilmicosin, and tildipirosin, primarily used in animals^13^. This difference in substrate spectrum is largely attributed to their different catalytic mechanisms. The Ere-type employs a catalytic histidine to generate a deprotonated water molecule for attacking the ester bond, whereas the Est-type utilizes a catalytic serine^14,15^. The Est-type, belonging to the alpha/beta hydrolase family, typically uses a catalytic triad (serine-histidine-aspartic acid), where histidine and aspartic acid activate the serine^16,17^. However, while the catalytic triad is present in EstT, its function has not been experimentally validated through mutagenesis, and the relationship between its structure and function is still unknown.

The current understanding of the distribution and transmission of EstT-type macrolide esterases is notably limited. This lack of comprehensive knowledge represents a barrier to the development of effective countermeasures against antibiotic resistance, thereby elevating the potential for unforeseen outbreaks of resistant bacterial strains with substantial public health ramifications. In this context, our study focuses on EstT-type macrolide esterase-EstX, which is hypothesized as macrolide esterase and not biochemically verified. We conducted an investigation of its enzymatic properties, including substrate spectrum and catalytic mechanisms. Furthermore, we explored its genomic context and assessed its global distribution, aiming to elucidate the driving forces behind its dissemination. The study of EstX’s catalytic mechanisms and modes of transmission provides critical insights, potentially informing strategies to mitigate the spread of antibiotic resistance genes.

## Materials and methods

### Gene synthesis, protein expression, and purification

Gene encodes EstX (genbank ID: PP210101.1, genbank protein ID: WVE62698.1) were synthesized into pET28a vector with 6X-His tag on protein C-terminal. After recombinant plasmid pET28a-estx was transformed into *E.coli* BL21 (DE3) (Thermo Scientific™, catalog number: EC0114), the cells were grown in liquid Luria-Bertani medium supplemented with 100 μg/mL kanamycin. When OD_600nm_ reached 0.6-0.8, 0.5 mM IPTG (Isopropyl β-d-1-thiogalactopyranoside) was added into the culture medium, followed by overnight cultivation at 25 °C. Then, cells were collected, resuspended with 20 mM Tris–HCl (pH = 7.0), and disrupted by sonification. After removing cell debris by centrifugation at 4 °C 10,000×*g* for 20 min, the supernatant was loaded onto the HisTrap column and EstX was purified by immobilized metal affinity (IMAC). Collected set fractions were dialyzed against 20 mM Tris–HCl (pH = 7.0) buffer 3 times and kept at 4 °C for further use. The protein purity was determined with sodium dodecyl sulfate–polyacrylamide gel electrophoresis (SDS–PAGE), and protein concentration was determined by using 280 nm absorbance and predicted calculated molar absorption coefficients.

### Biochemical characterization of EstX

Enzyme activity was determined using *p*-Nitrophenyl butyrate (*p*-NPB) as the substrate. 10 μL purified enzyme solution (0.8 mg/mL) was added to 990 μL of the substrate solution (2 mM *p*-NPB, 0.1% Triton X-100, and 20 mM Tris–HCl pH 7.0), and the reaction was carried out at 37 °C for 20 min. The reaction was stopped by adding methanol to 50% final concentration. The absorbance of OD_405nm_ was recorded using a UV spectrophotometer with heat-deactivated enzymes added substrate solution as the control. Enzyme activity was defined as the amount of enzyme that releases 1 µmol of *p*-Nitrophenol (*p*-NP) per minute under the specified conditions.

In order to investigate the optimal temperature for EstX, enzyme activity assays were conducted at different temperatures, specifically, 0, 10, 20, 30, 40, 50, 60, 70, 80, 90, and 100 °C. Meanwhile, to assess the enzyme’s optimal pH conditions, enzyme activity was evaluated at varying pH levels. To determine EstX’s thermostability and pH stability, EstX was incubated at corresponding temperature or pH conditions for 1 h, and residual enzyme activity was detected^18,19^.

### Minimum inhibitory concentration analysis

Minimum inhibitory concentration (MIC) analysis was conducted using the broth microdilution method and selected antibiotics included 13 macrolides (erythromycin, roxithromycin, clarithromycin, azithromycin, tulathromycin, josamycin, spiramycin, tylosin, tildipirosin, tilmicosin, medemycin, leucomycin A5, and acetylspiramycin), quinolones (ciprofloxacin, levofloxacin), β-lactams (amoxicillin, cefoxifin, cefotaxime), chloramphenicols (florfenicol), aminoglycosides (kanamycin), lincosamides (clindamycin, lincomycin) and tetracyclines (terramycin, doxycycline). Overnight cultures of *E. coli* were inoculated at a 1:2000 dilution into Mueller–Hinton (MH) broth, which was supplemented with 2-fold serial dilutions of antibiotics. Following an 18–20 h incubation at 37 °C, the absorbance of OD_600nm_ was measured, and the MIC was determined as the lowest concentration of antibiotic that resulted in the inhibition of bacterial growth.

### ESI-MS analysis and inhibition zone analysis of hydrolyzed macrolide antibiotic

100 μL purified EstX (0.8 mg/mL) was added into 900 μL, 50 μg/mL macrolide antibiotic solution. The reaction mixture was incubated at 40 °C for 1 h and 200 μL methanol was added to stop the reaction. ESI-MS was then employed to study if the macrolide antibiotic was successfully degraded. The mass spectrometry scan range was set at 100–1000*m*/*z*, with a curtain gas pressure of 40 psi, ion source gas 1 at 30 psi, ion source gas 2 at 30 psi, ESI^+^ (Electrospray Ionization) voltage at 5500 V, ESI^−^ voltage at 4500 V, and drying gas temperature and flow rate maintained at 450 °C and 5 L min^−1^, respectively.

Inhibition zone analysis was used to analyze the antimicrobial activity of the macrolide antibiotic before and after enzymatic hydrolysis. *Staphylococcus aureus* ATCC 25923, which was susceptible to macrolide antibiotics, was used as the test strain. A 20 μL overnight culture of *Staphylococcus aureus* was inoculated into 20 mL of liquid LB broth containing 1.5% agar at 40 °C. After thorough mixing, the mixture was poured into 9 cm Petri dishes. Once the agar solidified, 4.5 mm diameter wells were created using a hole punch, and subsequently, 40 μL of either unhydrolyzed or hydrolyzed macrolide antibiotics (50 μg/mL) were added. The Petri dishes were then incubated at 37 °C for 24 h, and the size of the inhibition zones was measured.

### Structural prediction of EstX

The 3D structure of EstX was predicted using Colabfold with the following parameter settings: number_recycles 50, max_msa 512-1024, number_relax 5, rank_plddt, and number_models 5^20^. Subsequently, the protein structure was visualized and analyzed in PyMOL^21^. The binding pocket on the EstX model was predicted using prankweb (https://prankweb.cz/)^22^. Molecular docking between EstX and tylosin was carried out using Libdock, and the docking result with the lowest Gibbs free energy was selected for subsequent analysis^23,24^.

### Site-directed mutagenesis

To further investigate EstX catalytic activity, site-directed mutagenesis S102A, D233A, and H261A were conducted by Phusion Site-Directed Mutagenesis Kit (Sigma). The mutated plasmid was transformed into *E. coli* and then sequenced to confirm. The procedures for plasmid construction, protein expression, protein purification, and enzyme activity assessment were consistent with those employed for EstX.

### Identification of EstX proteins from the bacterial database

We employed the NCBI bacterial genome database to investigate the distribution of the EstX protein across bacterial genomes. Briefly, a total of 1.3 million bacterial genomes were downloaded from the NCBI reference database (July 2023), and subsequently, protein sequences from each genome were compiled into a fasta file. Then Diamond was used to perform protein sequence similarity analysis using EstX as the query sequence. Through this process, we identified bacterial genomes that had EstX (100% identity and 100% coverage). In this study, to avoid introducing false positives, only genes encoding proteins with 100% sequence similarity to EstX were considered. Meanwhile, information such as collection data, host, and location for each identified genome was also summarized.

### Genomic context analysis

We analyzed the Pfam families of proteins surrounding EstX, including the 10 proteins upstream and 10 proteins downstream. First, we extracted 20 protein sequences around EstX from each individual genome, and subsequently, hmmer used to identify each protein’s Pfam family (*E*-value: 1e−10, minimum matched length: 50). Second, we summarized the occurrences of Pfam families across different bacterial genomes and only pfam family with a >10% occurrence rate was kept. Finally, we employed a heatmap to visually represent the presence of Pfam protein families within various genomic contexts. The visualization of linkages between different representations of these analyses was generated using the clinker online website (https://cagecat.bioinformatics.nl/).

### Statistics and reproducibility

Data were presented as means ± standard deviation (SD) as indicated in the figure captions. To compare different experimental groups, the *t*-Student test was employed. All analyses were conducted in R-studio, and all experiments had a minimum of three biologically independent replicates.

## Results

### EstX is a novel Est-type macrolide esterase

EstX was first found in *E. coli* from pigs, sewage, and people in the 1980s, while its function was unknown^25,26^. Our analysis showed that EstX exhibited low sequence similarity with experimentally characterized proteins, including 44% with EstT, 36.84% with aclacinomycin methylesterase RdmC, 32.86% identity with rhodomycin D methylesterase DauP, and 32.40% identity with rhodomycin D methylesterase DnrP (Supplementary Table 1). It has been suggested as a macrolide esterase based on its 44% sequence homology with the recently reported macrolide esterase EstT^4,13^. As the function of EstX had not yet been experimentally studied, we synthesized this gene in our laboratory to study its function.

**Fig. 1:**
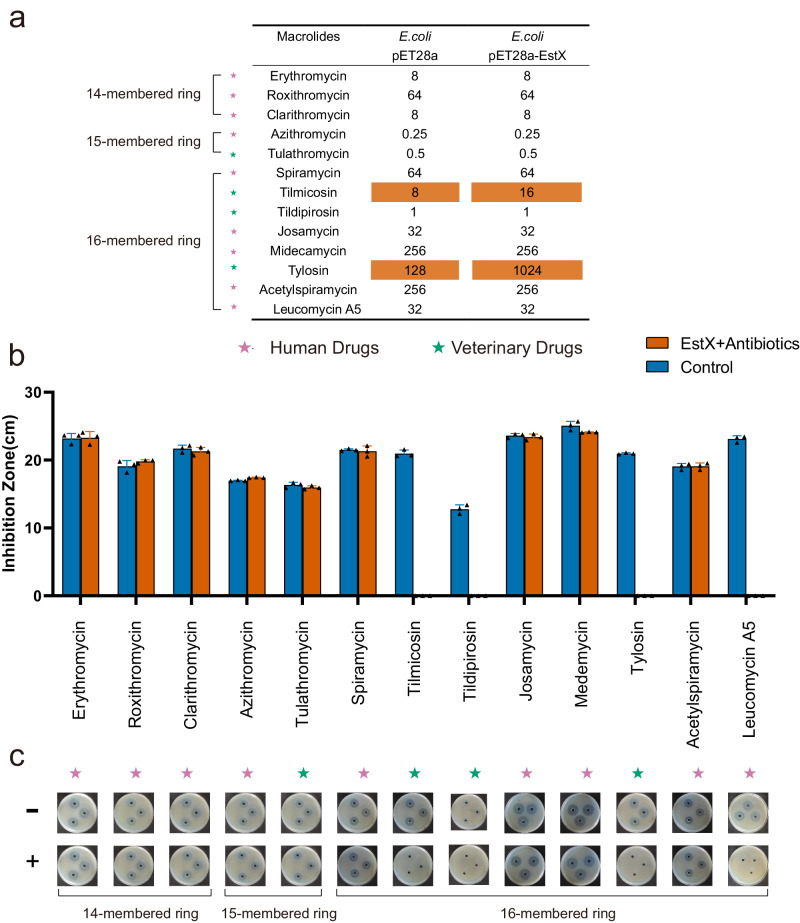
Minimum inhibitory concentration (MIC) analysis of EstX-carrying *Escherichia coli* and macrolide esterase function confirmation using inhibition zone analysis. **a** MIC analysis of 13 macrolides antibiotics for *Escherichia coli* carrying either pET28a or pET28a-EstX. MIC values were determined using the broth microdilution method. **b** Plate inhibition zone sizes of 13 macrolides antibiotics before and after EstX hydrolysis. Agar plates were supplemented with *Staphylococcus aureus* ATCC 25923 to evaluate the impact of EstX-mediated hydrolysis on antibiotic activity. Data are presented as the mean ± SD of independent experiments. *n*  =  3 biologically independent experiments. **c** Plate inhibition zone analysis of 13 macrolide antibiotics against *S. aureus* ATCC 25923, both before and after EstX hydrolysis.

For 14-membered, 15-membered, and most 16-membered macrolide antibiotics, in addition to β-lactams, chloramphenicol, aminoglycosides, lincosamides, and tetracyclines, no alteration in the MICs was observed in *E. coli* carrying the pET28a-EstX plasmid (Fig. 1a and Supplementary Table 2). However, the MICs for tilmicosin and tylosin in the presence of EstX were 16 and 1024 μg/mL, respectively. These values were 2-fold and 8-fold higher compared to those without the pET28a vector, indicating that EstX enhanced *E. coli*’s resistance to tilmicosin and tylosin (Fig. 1a). This observation differed from the MIC changes observed in *E. coli* carrying the EstT, which showed increased MICs for tilmicosin, tildipirosin, and tylosin.

To understand the underlying mechanisms of EstX, we expressed and purified EstX. SDS–PAGE showed that EstX was solubly expressed in *E. coli* (Supplementary Fig. 1). After IMAC purification, EstX’s showed >90% purity, which was sufficient for subsequent biochemical analysis. Its molecular weight was estimated as 33 kDa on SDS-PAGE, consistent with its predicted molecular weight (Supplementary Fig. 1, Fig. 1b). Meanwhile, EstX showed three different bands in Native-PAGE and indicated it might function as dimer or multimer (Supplementary Fig. 2). We then investigated the impact of EstX-mediated hydrolysis on the antimicrobial activity of macrolide antibiotics using inhibition zone assays. After overnight enzymatic treatment, the inhibition zones for tylosin, tilmicosin, tidipirosin, and leucomycin A5 disappeared, indicating that EstX can inactivate these four antibiotics (Fig. 1c). This result presented an inconsistency with the MIC results. If EstX was capable of degrading four antibiotics, one would expect differences in the MIC values for four antibiotics in *E. coli* carrying the pET28a-EstX. However, differences in MIC values were only observed for tylosin and tilmicosin, while the MICs for leucomycin A5 and tidipirosin remained unchanged.

### EstX hydrolyzes 16-member ring macrolide antibiotics used in both humans and animals

Inhibition zone analysis revealed complete degradation of the antibiotics by EstX, raising the question of why corresponding changes in the MIC phenotype were not observed. We hypothesized that the resistance changes induced by EstX might be subtle, resulting in no alterations in MIC values. To investigate this, we calculated the IC50 values for the four antibiotics. We observed an increase in the IC50 values for *E. coli* carrying the EstX plasmid against all four antibiotics. For instance, the IC_50_ for tylosin increased from 17.47 to 72.97 μg/mL, tildipirosin from 0.20 to 0.27 μg/mL, tilmicosin from 2.05 to 3.26 μg/mL, and leucomycin from 0.96 to 2.04 μg/mL (Supplementary Fig. 3). Therefore, the changes in IC_50_ indicated an alteration in the antibiotic resistance of the bacteria carrying EstX.

**Fig. 2:**
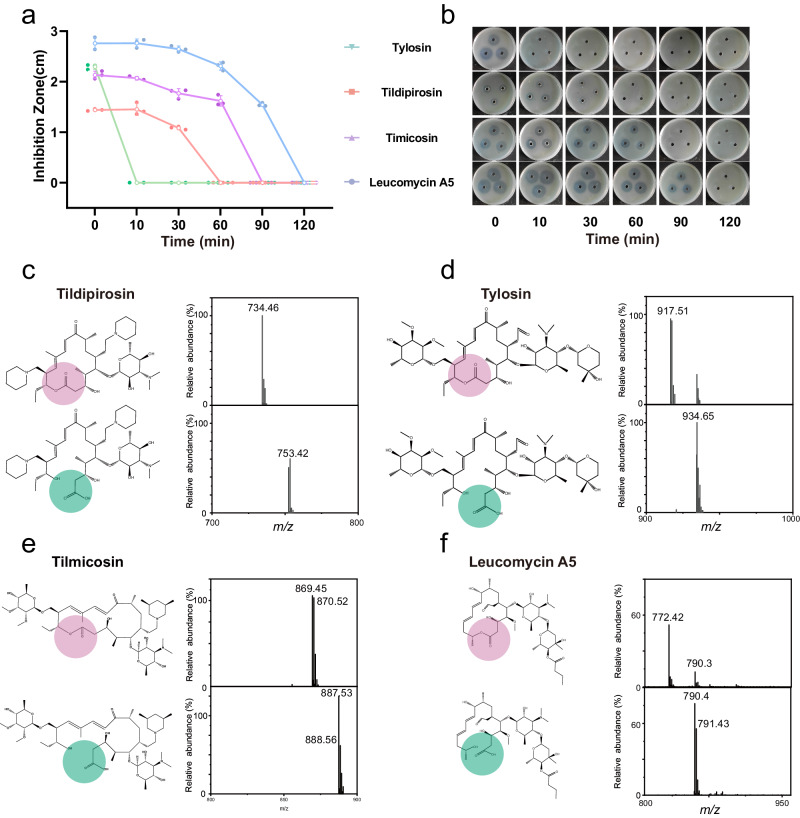
Time-dependent plate inhibition zone analysis and electrospray ionization mass spectrometry (ESI–MS) analysis of the hydrolyzed products by EstX. **a** Plate inhibition zone size analysis of four macrolide antibiotics (tylosin, tildipirosin, tilmicosin and leucomycin A5) following hydrolysis by the EstX enzyme at various time points (0, 10, 30, 60, 90, 120 min). The diameter of inhibition zones was measured to evaluate the time-dependent degradation of the antibiotics by EstX. Data are presented as the mean ± SD of independent experiments. *n*  =  3 biologically independent experiments. **b** Plate inhibition zone analysis of four macrolide antibiotics against *Staphylococcus aureus* ATCC 25923 following hydrolysis by EstX. **c**–**f** Electrospray ionization mass spectrometry (ESI–MS) analysis of the products before and after EstX hydrolysis of tylosin, tildipirosin, tilmicosin, and leucomycin A5. The hydrolysis sites of the macrolide antibiotics are indicated by circles, where red indicates before hydrolysis and green indicates after hydrolysis.

*E. coli* carrying the EstX plasmid exhibited increased resistance to all four antibiotics, but differences in MIC were only observed for tylosin and tilmicosin, leading us to investigate the underlying cause of this phenomenon using time-dependent inhibition zone analysis. The results revealed the minimum time required for the disappearance of the inhibition zones for each antibiotic: tylosin (10 min), tidipirosin (60 min), tilmicosin (90 min), and leucomycin A5 (120 min). Thus, the rate of degradation by EstX for these antibiotics follows the order: tylosin > tidipirosin > tilmicosin > leucomycin A5 (Fig. 2a and b). Therefore, although EstX can degrade all four antibiotics, the degradation efficiencies were different. A slower degradation rate might prevent the rapid breakdown of antibiotics that enter the bacterial cells, potentially resulting in unchanged MIC values. This explains the observed differences in MICs for *E. coli* with pET28a-EstX plasmid when different antibiotics are used.

EstX enzyme activity studies showed that its optimal temperature was 40 °C, with activity decreasing to 32% and 17% at 30 and 50 °C, respectively. In addition, EstX was stable in the temperature range of 0–40 °C and could maintain 78–100% activity after 1 h of incubation, but the activity dramatically decreased to 13% when exposed to 50 °C (Supplementary Fig. 4). The instability of EstX above 50 °C was further confirmed through dynamic light scattering (DSL) analysis, indicating that EstX started to form aggregates at 49.3 °C, and by differential scanning fluorimeter (DSF) results, showing a melting temperature of 51.8 °C (Supplementary Fig. 5). The optimal pH for EstX was 7, with activity dropping to 51% at a pH of 8. In both acidic (pH 3–6) and alkaline (pH 9–11) buffers, EstX activity remained below 20%. In addition, EstX exhibited optimal stability at pH 7, with a decrease observed at both higher and lower pH. Therefore, EstX exhibited optimal activity at pH 7.0 and 40 °C, showing relative stability in environments below 50 °C or at neutral pH.

Though EstX could inactivate four 16-membered ring macrolides in the inhibition zone experiment, little is known about how EstX alters the macrolide structure. After enzyme treatment, the molecular weights of four antibiotics changed, tylosin (917.51*m*/*z* increased to 934.65*m*/*z*), tildipirosin (734.46*m*/*z* increased to 753.42*m*/*z*), tilmicosin (869.45*m*/z increased to 870.53*m*/*z*) and leucomycin A5 (772.42*m*/*z* increased to 790.40*m*/*z*) (Fig. 2c–f). The molecular weight of all four antibiotics increased by 18*m*/*z* after hydrolysis, suggesting the hydrolysis of the ester bond. However, for other macrolides antibiotics with 14-membered ring, 15-membered ring, and 16-membered ring macrolides, the molecular weight remained unchanged after treating with EstX (Supplementary Fig. 6).

### EstX utilizes catalytic triad for ester bond hydrolysis

**Fig. 3:**
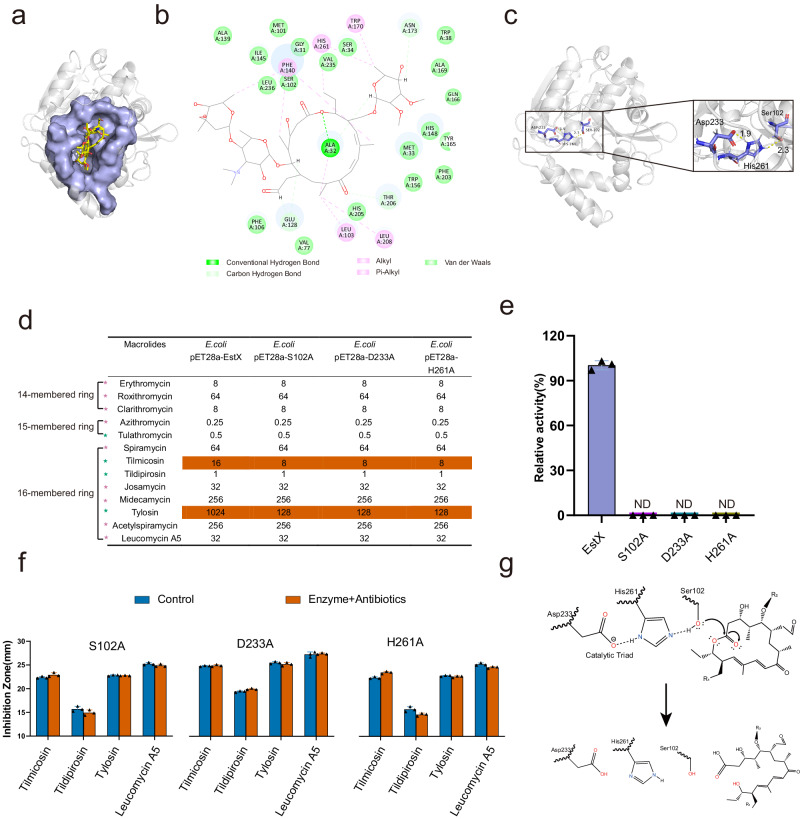
Catalytic mechanism of EstX. **a** Molecular docking of tylosin with the predicted EstX structure. Molecular docking was performed using Libdock and then visualized using PyMOL software. **b** Two-dimensional diagram illustrating interactions between the predicted EstX structure and tylosin. Different bonds are represented by dashed lines of different colors. **c** Predicted EstX structure highlighting the catalytic triad composed of Ser102, Asp233, and His261. Hydrogen bonds are indicated by dashed lines, and numbers represent the lengths of the hydrogen bonds (Å). **d** Minimum inhibitory concentration (MIC) analysis of EstX mutations against 13 macrolide antibiotics. **e** Enzyme activity analysis of EstX mutations. Esterase activity was determined using *p*-Nitrophenol as substrate. Data are presented as the mean ± SD of independent experiments. *n*  =  3 biologically independent experiments. **f** Plate inhibition zone sizes of products derived from four macrolide antibiotics (tylosin, tildipirosin, tilmicosin, leucomycin A5) before and after hydrolysis by EstX mutations (S102A, D233A, and H261A). Data are presented as the mean ± SD of independent experiments. *n*  =  3 biologically independent experiments. **g** Proposed catalytic mechanism of EstX, which hydrolyzes the ester bond of tylosin, resulting in the ring-opening of the macrolide.

Currently, the catalytic mechanism of Est-type macrolide esterases has not been well investigated. To address this, we used alphafold2 to predict EstX structure, and a PLDTT score (97.3) indicated the reliability of the predicted structure for further study (Supplementary Fig. 7). The molecular docking results between EstX and 4 macrolide antibiotics showed that van der Waals forces, conventional hydrogen bond, carbon–hydrogen bonds, alkyl bonds, and pi-alkyl bonds were the main interaction type (Fig. 3a and b; Supplementary Fig. 8 and Supplementary Table 3). The amino acids that interact with macrolide antibiotics were Gly31, Ser34, Ser102, Ala169, Asn173, Val235, and Leu236, which are located within the catalytic pocket (Supplementary Table 4). Among them, S102 was hypothesized as the catalytic amino acid because it was conserved in multiple sequence alignment and sat within the binding pocket. The spatial distances between S102 and macrolide ester bond were 3.18 Å (tylosin), 3.67 Å (tilmicosin), 3.12 Å (tildipirosin), and 3.86 Å (leucomycin A5), respectively, which were deemed sufficient for S102 to attack ester bond and perform the function (Supplementary Fig. 9).

EstX belongs to the alpha/beta hydrolase family, while the characteristic catalytic triad of this family has not been experimentally studied in macrolide esterases. In the predicted EstX structure, the catalytic triad was formed by Asp233-His261-Ser102, where Asp233 was linked to His261 by a 1.9 Å hydrogen bond, His261 was linked to Ser102 by a 2.3 Å hydrogen bond (Fig. 3c). To further confirm the catalytic triad, we constructed and purified mutations including S102A, D233A, and H261A, respectively (Supplementary Fig. 10). Firstly, The MICs of the *E coli* carrying S102A, D233A, and H261A were lower than that of the wild type EstX and showed no difference compared to *E coli* carrying the empty vector (Fig. 3d). The alteration in MIC values suggests that a mutation in any amino acid within the catalytic triad would result in the loss of protein activity. Secondly, enzyme activity assay using purified S102A, D233A, and H261A (with >90% purity) with p-NPB as a substrate showed that all three mutants lost activity (Fig. 3e and f). When a macrolide antibiotic was used as substrate and treated with purified S102A, D233A and H261A, the corresponding inhibition zone was similar to that of untreated macrolide antibiotics, indicating that mutants lost activities against macrolide antibiotics (Fig. 3f). Thus, the predicted protein structural information, point mutation MIC values, loss of activity, and inhibition zone results demonstrated that the catalytic triad in predicted EstX structure was crucial for its activity (Fig. 3g).

### *estX* carrying gene cascade spreads through mobile elements

**Fig. 4:**
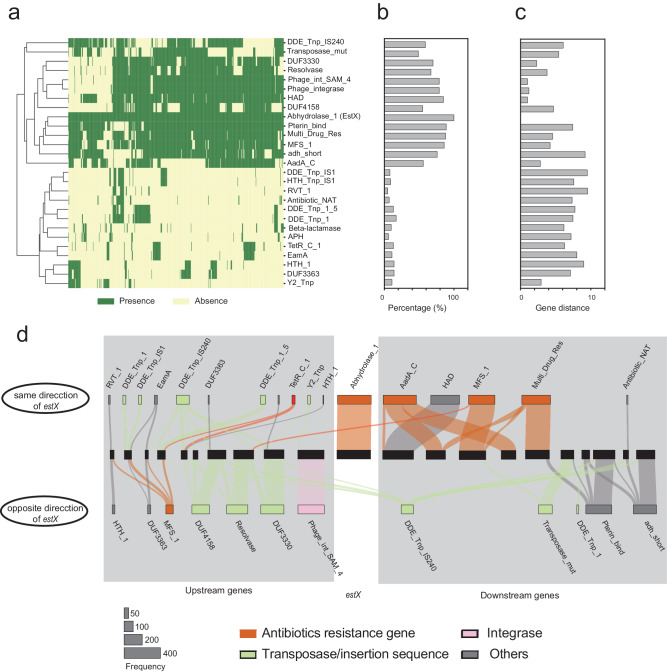
Pfam family information of genes around EstX. **a** Heatmap analysis illustrating the distribution of Pfam families within the 20 genes upstream and downstream of the *estX* gene. Green indicates the presence of the Pfam family, while light yellow indicates its absence. **b** Percentage distribution of Pfam families within the 20 genes upstream and downstream of the *estX* gene. **c** Gene distance between genes from different Pfam families and *estX*. **d** Frequency diagram showing the occurrence of various Pfam families in the genomic regions surrounding the *estX* gene. Pfam families related to antibiotic resistance are highlighted in red, those related to integrases are marked in pink, and those associated with transposases/insertion sequences are marked in green.

Investigating the gene families both upstream and downstream of *estX* can understand the transferability and co-occurring genes. Genes located upstream and downstream of *estX* belong to pfam families associated with drug resistance (e.g., MFS_1, AadA_C, Beta-lactamase, TetR_C_1, EamA, Multi_Drug_Res), mobile genetic elements (e.g., DDE_Tnp_1, DDE_Tnp_IS1, DDE_Tnp_IS240, DDE_Tnp_1_5, Y2_Tnp, DUF4158, Resolvase, DUF3330, Transposase_mut) and other functions (e.g., RVT_1, DUF3363, HAD, HTH_1, Pterin_bind and adh_short) (Fig. 4a). Heatmap cluster analysis divided the pfam families into two groups: a high-frequency group (55–100%) and a low-frequency group (5–49%) (Fig. 4b). Among the high-frequency proteins, the average distances between *estX* and genes from Phage_int_SAM_4, HAD, Phage_integrase, DUF3330, and AadA_C were 1, 1.01, 1.20, 2.31 and 2.82, respectively, indicating that they were likely to be adjacent to *estX* (Fig. 4c).

In the genomic region flanking the *estX* gene, a directional gene cascade has been observed, forming a coherent cluster with other protein-coding genes. This cluster includes the *aadA* gene from the AdaA_C family, conferring aminoglycoside resistance; a HAD family gene with unknown function; the *cmlA1* gene from the MFS-1 family, causing chloramphenicol resistance; and the *qacL* gene from the Multi_Drug_res family, associated with multidrug resistance (Fig. 4d). Within this gene cluster, The HAD gene is positioned first, and *cmlA1* occupies the third position. *aadA* genes appear in the 1st, 2nd, and 4th positions in the same orientation as *estX*, while *qacL* is found in the 2nd, 3rd, and 5th positions. This arrangement may be attributed to gene rearrangements within the *estX*-associated gene cluster.

The gene cascade surrounding *estX* is flanked on both sides by mobile elements associated with gene transfer, including type I integrases and transposases. Notably, a type I integrase is located opposite to *estX* in the first position, forming a complete integron. Additionally, genes associated with transposon, including transposase modulator proteins from the DUF3330 family, transposon resolvase from the Resolvase family, and transposase from the DUF4158 family, are present in the 2nd, 3rd, and 4th positions in the opposite orientation to *estX*. Other transposon-related families, such as Transposase DDE domain (DDE_Tnp_1, DDE_Tnp_IS1, DDE_Tnp_IS240, DDE_Tnp_1_5, Y2_Tnp, DUF4158, Resolvase, DUF3330, Transposase_mut), and Mutator transposable elements from the Transposase_mut family were also found in both sides of this gene cascade. Beyond integrases and transposases, 12.48% of *estX* distribution has been traced to plasmids, suggesting that, in addition to integrons and transposons, plasmids also play an important role in the dissemination of *estX*.

**Fig. 5:**
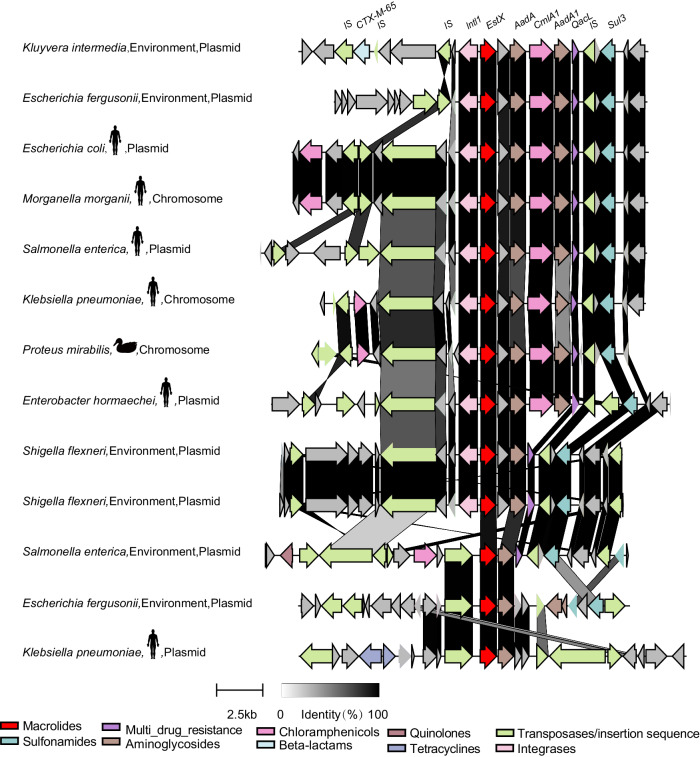
Genomic context linkage analysis of *estX*-carrying loci from representative bacterial species. Antibiotic resistance genes (ARGs), transposases, insertion sequences, and integrases are colored in different colors, while proteins with other functions are colored in gray. If two sequences from two loci share sequence similarity, they are linked with shadows that change color according to sequence similarity. To avoid introducing false positives, only genes encoding proteins with 100% sequence similarity to EstX were considered.

We next investigated the genomic context of *estX* across representative bacterial species, including *Kluyvera intermedia*, *E.coli*, *Morganella morganii*, *Salmonella enterica*, *K. pneumoniae*, *Proteus mirabilis*, *Enterobacter hormaechei*, *Shigella flexneri*, and *Escherichia fergusonii*. Our observations initially highlight interspecies horizontal gene transfer events, evidenced by the distribution of the gene cascade (EstX-HAD-AdaA-CmlA-AdaA-QacL) among various species such as *K. intermedia*, *Escherichia fergusonii*, *E.coli*, *M. morganii*, *S.enterica*, *K.pneumoniae*, *P. mirabilis*, and *E. hormaechei* (Fig. 5). This gene cluster is not only widely distributed but also exhibits a high degree of structural consistency and sequence homology. Furthermore, our analysis revealed gene dissemination between bacteria from different environmental origins. For instance, the gene clusters found in human-derived bacteria mirror those found in bacteria from animal and environmental sources, suggesting widespread transmission across different bacterial communities. Lastly, we noted instances of gene rearrangement and loss within the gene cascade containing *estX* during its transmission. A notable example includes the loss of the proteins *cmlA1* and *adaA1* in *Shigella flexneri*. These findings provide insight into the dynamic nature of gene transfer and the evolutionary pressures that shape bacterial pan-genomes.

### *estX* distribution across various pathogens worldwide

**Fig. 6:**
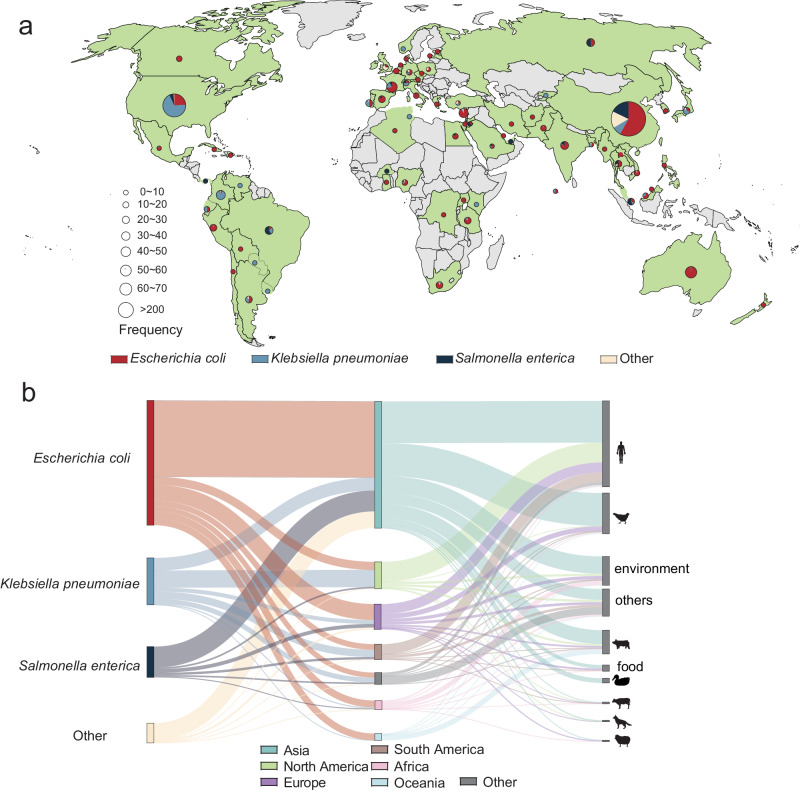
Geographical, ecological, and host distribution of *estX.* **a** World map showing the global distribution of *estX*-carrying bacteria. Countries with *estX*-carrying bacteria are colored green, while those without *estX*-carrying bacteria are colored gray. Countries with *estX*-carrying bacteria are colored green, while those without are colored gray. Each country is overlaid with a pie chart, where the size of each pie represents the number of *estX*-carrying bacteria, and the colors represent different *estX*-carrying bacteria species. **b** Sankey diagram depicting the distribution across continents and ecological niches of *estX*-carrying bacteria from various species. To minimize false positives, only genes encoding proteins with 100% sequence similarity to EstX were included.

A comprehensive survey identified 2259 bacterial strains from 74 countries harboring the *estX* gene. It is noteworthy that potential biases in the data could arise due to their reliance on isolates obtained from clinics or environmental samples, potentially impacting bacterial species, ecological niches, and geographic distribution. China exhibited the highest prevalence of *estX*, with 1026 strains (45.42%), followed by the USA with 248 strains (10.98%), and subsequent contributions from Australia (*n* = 68, 3.01%), France (*n* = 51, 2.26%), and Lebanon (*n* = 49 2.17%). Among the *estX*-bearing strains, the majority belonged to pathogenic or opportunistic pathogens, with *E. coli* (1269 strains, 56.18%), *K. pneumoniae* (477 strains, 21.12%), and *S. enterica* (477 strains, 21.12%) collectively constituting over 91.14% of the total strains analyzed (Fig. 6a). The presence of *estX* in clinical isolates underscores its potential association with pathogenicity in both humans and animals.

A notable variability in bacterial distribution was observed at both the country and continental levels. For instance, 5 countries (China, Australia, France, Lebanon, and India) exhibited dominance of *E. coli*, while 2 were predominated by *K. pneumoniae*, and 2 by *S. enterica*. Interestingly, each continent showed a distinct prevalence pattern: Asia led with *E. coli* (60.30%), North America with *K.pneumoniae* (63.37%), and Africa with *E. coli* (76.04%). These patterns highlight the distinct ecological niches of *estX*-bearing bacteria across different regions (Fig. 6b).

The ecological diversity of *estX* is evident from its presence in various environments, including host-associated environments (2015 strains, 89.20%), the general environment (293 strains, 12.97%), and food sources (63 strains, 2.79%). Within host-associated environments, human-related strains predominated, but a substantial presence was also noted in animals such as pigs (*n* = 241), chickens (*n* = 414), ducks (*n* = 48), cows (*n* = 18), dogs (*n* = 16) and sheep (*n* = 15) (Fig. 6b). These findings emphasize the role of host-associated environments as primary reservoirs for *estX*. Moreover, *estX* ‘s presence in diverse environments, ranging from livestock farms to sewage treatment plants and rivers, and intensive care unit wards, suggests its potential as a reservoir for drug-resistant genes. Additionally, the detection of *estX* in food products like chicken and beef surfaces further complicates the public health implications of its spread.

### Emergence timeline of estX-carrying bacteria

**Fig. 7:**
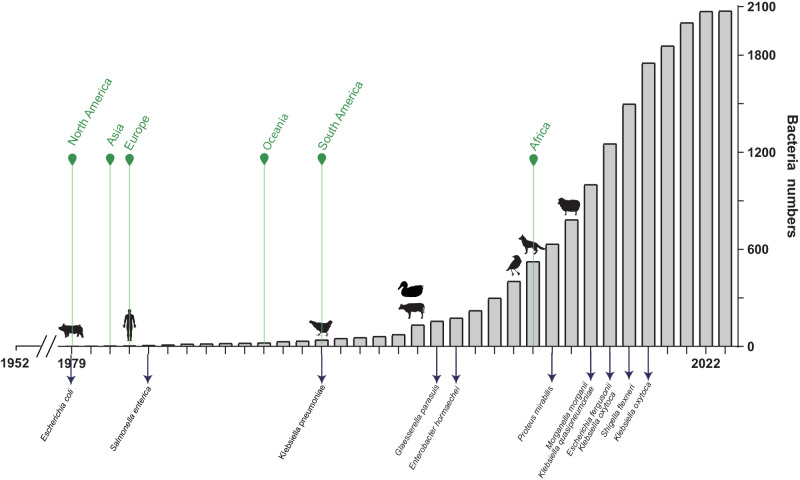
Timeline of the emergence of *estX*-carrying bacteria from 1979 to 2023. Green arrows indicate the first appearance of *estX*-carrying bacteria on each continent. Black arrows indicate the first discovery of *estX* in each bacterial species. The human or animal icon on each bar represents the first discovery of *estX*-carrying bacteria within that host. To reduce false positives, our analysis focused exclusively on bacteria-carrying genes encoding proteins with 100% sequence identity to EstX.

The emergence timeline indicated that bacteria carrying the *estX* gene were first found in 1979, ~27 years after the use of macrolides (1952). We observed a gradual increase in the detection of *estX*-carrying bacteria from 1979 through 2023, with a notably sharp rise from 2006 onwards. This escalation can be attributed to advancements in sequencing technologies facilitating the discovery of gene carriers. The timeline of *estX* discovery began with its detection in *E. coli* in 1979. Subsequent discoveries were in *S. enterica* in 1990, *K.pneumoniae* in 2002, and several other species, including *Glaesserella parasuis* in 2008 and *E. hormaechei* in 2009. More recent identifications include *P. mirabilis* in 2014, *M. morganii* and *Klebsiella quasipneumoniae* in 2016, *E. fergusonii* and *Klebsiella oxytoca* in 2017, *Shigella flexneri* in 2018, and another strain of *K. oxytoca* in 2019 (Fig. 7). Notably, the majority of bacteria hosting the *estX* are associated with human and animal diseases, underscoring the gene’s association with pathogenicity.

The geographical discovery of *estX*-carrying bacteria aligns with the historical usage of tylosin. North America was the first reported continent in 1979, followed by Asia in 1984 and Europe in 1989. Oceania followed in 1999, South America in 2002, and finally Africa in 2013. Meanwhile, regarding the hosts, the *estX* gene was initially found in pigs in 1979, then in humans in 1989, in chickens in 2002, and subsequently in ducks and cattle in 2007. Later discoveries included birds in 2012, dogs in 2013, and sheep in 2015. It is imperative to highlight that the hosts of *estX*-carrying bacteria encompass not only livestock and poultry but also pets and wildlife.

*estX* poses a threat not only to existing natural macrolide antibiotics but also to semisynthetic antibiotics in the development stage that have not yet been utilized. The discovery of natural macrolides, such as tylosin and leucomycin A5, dates back to the 1950s. Tylosin has been predominantly utilized in veterinary medicine and as an agent for growth promotion, whereas leucomycin A5 has seen significant use in clinical settings. Approximately two decades following their introduction, in 1979, *estX* was identified in *E. coli* strains found in pigs. This timeline suggested a lag in the emergence of resistance following antibiotic use.

## Discussion

The global dissemination of *estX* is likely facilitated by various mobile genetic elements, including integrons, transposons, and plasmids. Class I integron can acquire *estX* and other ARG genes (including *aadA*, *cmlA1*, and *qacL*) to form a tightly linked gene cassette which is coregulated and coexpressed to respond more rapidly to antibiotic stress^27^. This kind of gene cassette structure enables its prevalence and persistence in the environment, even in sub-inhibitory concentration^27,28^. Class I integron do not encode enzymes enable horizontal gene transfer between bacteria, it often present in plasmid or transposons, which contributes to horizontal gene transfer between same or different bacterial species^29^. Gene transfer using different mobile elements and collaborations between mobile genetic elements (integron and transposon, integron and plasmid, integron plasmid and transposon) can accelerate the transfer between bacteria and, therefore, enabling *estX*’s wide distribution globally^30^. Notably, this gene cassette formed by a Class I integron, observed in the context of *estX*, differs from the genomic context of *estT*, which primarily propagates through transposases and plasmids. Therefore, the rapid dissemination of Est-type macrolide esterases can be attributed to their transmission via a variety of genetic elements, including integrons, transposons, and plasmids.

Est-type macrolide esterase, a class of resistance genes discovered in 2023, is of significance. Historically, the most well-known macrolide esterases have been of the Ere-type, known for their ability to degrade 14–15-membered ring macrolide antibiotics. In contrast, the Est-type macrolide esterase comprises only two members, EstT and EstX, with 109 and 2259 identical proteins, respectively. The enzymatic properties of EstX, optimal at 40 °C and pH 7.0, suggest its ecological origins are predominantly associated with hosts. This is further supported by its presence in host-associated pathogenic bacteria across 74 countries spanning six continents. The extensive distribution and spread of *estX* over the past 50 years can likely be linked to the widespread use of veterinary antibiotics like tylosin, commonly used as growth promoters, and the clinical use of the drug leucomycin A5, which has exerted selective pressure. Interestingly, the two antibiotics that EstX is capable of degrading were introduced and utilized after EstX became prevalent. This sequence of events underscores the threat EstX poses not just to existing macrolide antibiotics, but also to the efficacy of newer macrolides still in the developmental stages. Considering single-point mutations can change the substrate specificity of the antibiotic-resistant gene, the potential risks Est-type genes pose, necessitating further in-depth research and careful evaluation.

This study uncovers EstX’s ability to hydrolyze 16-membered ring macrolides, encompassing human-use antibiotics, with site-directed mutagenesis revealing a typical catalytic triad mechanism in the alpha/beta hydrolase superfamily. The research also indicates the potential for EstX’s global spread of bacterial pathogens via various mobile genetic elements. Considering the presence of other EstX-like macrolide esterases in nature, it becomes imperative to conduct future research and surveillance focusing on novel EstX-type esterases. This is especially critical for those variants capable of degrading human-use macrolides, as they pose a threat to the effectiveness of clinical macrolide antibiotics. A comprehensive understanding and monitoring of these enzymes are essential in laying the groundwork for the prevention and control of macrolide antibiotic resistance.

### Supplementary information

Supplementary Information.pdf

Description of Additional Supplementary Files

Supplementary Data

## Data Availability

The data that support the findings of this study are available in the Supplementary Data file.
