# Supplementary material for: The global distribution of the macrolide esterase EstX from the alpha/beta hydrolase superfamily
Source: Commun Biol. 2024 Jun 28;7:781. doi: 10.1038/s42003-024-06473-2 (PMC11214618; doi:10.1038/s42003-024-06473-2)
Supplement: Supplementary file 1 — Supplementary Information.pdf [file 42003_2024_6473_MOESM1_ESM.pdf]

The global distribution of the novel macrolide esterase EstX from the alpha/beta hydrolase superfamily

Jiafu Lin[1*], Hua Lv[1*], Tiantian Wang[1], Hongkun Tao[1], Yi Zhong[1], Yang Zhou[1], Yibo Tang[1], Feng Xie[1], Guoqing Zhuang[3], Changwen Xu[1], Yiwen Chu[1], Xinrong Wang[1], Yongqiang Yang[2†] and Tao Song[1†]

1: Antibiotics Research and Re-evaluation Key Laboratory of Sichuan Province, Sichuan Industrial Institute of Antibiotics, School of pharmacy, Chengdu University, 610106, Chengdu, China

2: Center of Infectious Diseases, Center for Pathogen Research, West China Hospital, Sichuan University, Chengdu, China

3: Sichuan Academy of Forestry, Chengdu, 610081, Sichuan, China

*These authors contributed equally to this work.

†Corresponding author: Yongqiang Yang and Tao Song

E-mails: yangyq76@scu.edu.cn and songtao@cdu.edu.cn

Supplementary figure 1

SDS-PAGE analysis showing the purification of EstX, lanes are labeled for soluble protein of *E.coli* pET28a induced by isopropyl β-D-thiogalactoside (IPTG) (1), inclusion protein of *E.coli* pET28a induced by isopropyl β-D-thiogalactoside (IPTG), and purified EstX after immobilized metal affinity (IMAC)

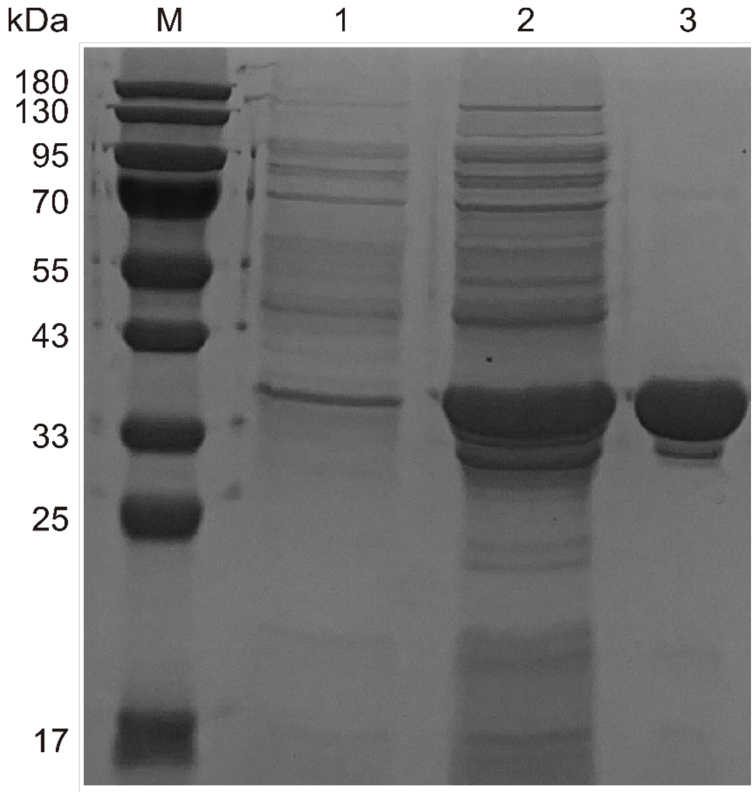

Supplementary figure 2

a: Flowchart illustrating the investigation of EstX oligomeric state through Native-PAGE, protein recovery, and activity validation. Briefly, after conducting Native-PAGE for EstX, prominent bands were identified and excised using a scalpel, followed by immersion in a 20 mM Tris-HCl buffer (pH 7.0) for 8 hours and subsequent centrifugation to collect the supernatant. Esterase activity was assessed using p-Nitrophenol as a substrate, and protein bands from the recovery process were analyzed via SDS-PAGE. b: SDS-PAGE analysis of purified EstX. c: Native-PAGE analysis of purified EstX, with bands of interest highlighted in the blue box and labeled as R1, R2, R3, and R4. EstX-DTT indicates the addition of a final concentration of 10 mM DTT to the samples. D: SDS-PAGE analysis of purified EstX and recovered protein samples (R1, R2, R3, and R4), with green checkmarks indicating esterase activity and red crosses indicating lack thereof.

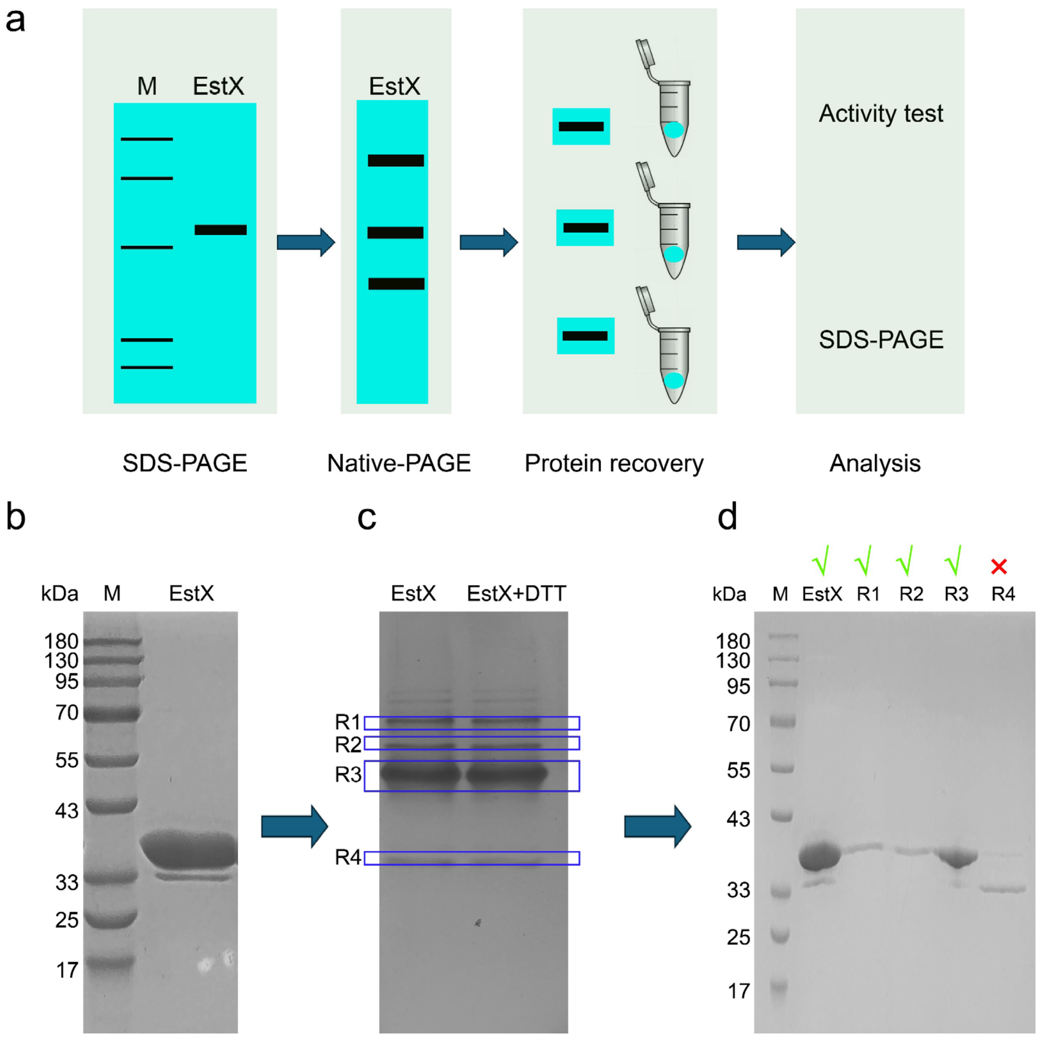

Supplementary figure 3

Figure a to d show the IC50 values of 4 macrolide antibiotics (Tylosin, Timicosin, Tildipirosin, Leucomycin A5) against (*E. coli* pET28a and *E. coli* pET28a-EstX). IC50 value indicates the antibiotic concentration required to inhibit 50% of bacterial growth. “*” indicates that there is a significant difference in IC50 values between the *E. coli* pET28a and *E. coli* pET28a-EstX. Data are presented as the mean ± SD of independent experiments. n = 3 biologically independent experiments. Statistical significance was assessed by a two-tailed unpaired t-student test (*P<0.05, **P<0.01, ***P<0.001).

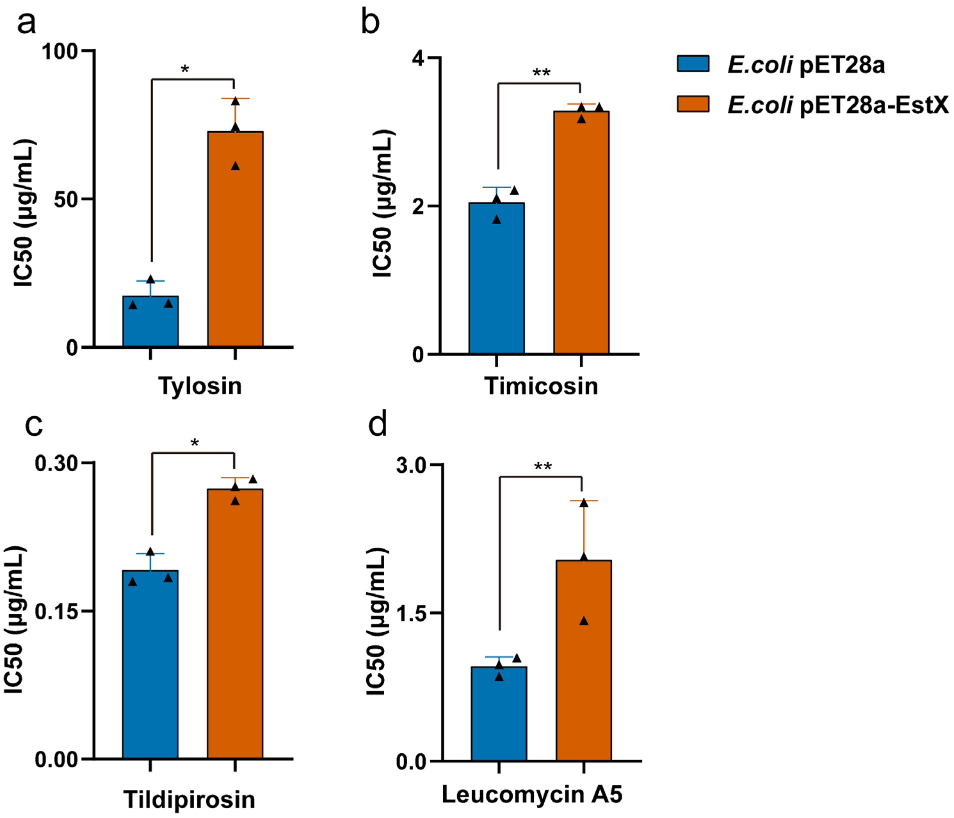

Supplementary figure 4

Figure a: shows that the optimal temperature for EstX is 40°C. Figure b shows that EstX has high thermal stability at 0-40°C, but rapidly loses activity at 50°C and 60°C. Figure c shows that the optimal pH for EstX is 7.0. Figure d shows that EstX has high pH stability in the range of pH 6.0-8.0, but reduces activity at pH 5.0 and pH 9.0. Data are presented as the mean ± SD of independent experiments. n = 3 biologically independent experiments.

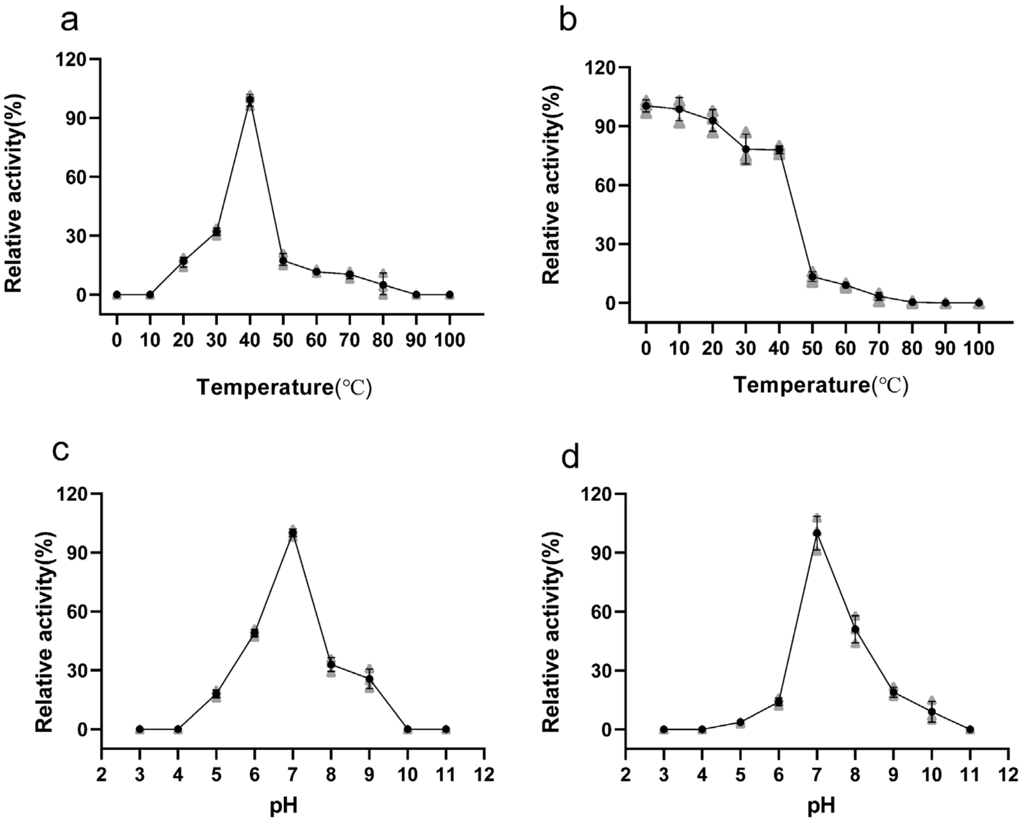

Supplementary figure 5

Thermal unfolding experiments of the purified EstX were carried out by NanoTemper PR. 0.8 mg/mL purified EstX is filled into the capillaries by capillary force action and then placed into the instrument. The capillaries are single-use and therefore no equilibration or cleaning is required. The capillaries were heated from 20 to 90 °C with a heating rate of 1 °C /min and the changes in the fluorescence ratio (OD_{350nm}/OD_{330nm}) and scattering score was monitored to determine the Tm (melting temperature) and Tagg (temperature that protein starts to form aggregates).

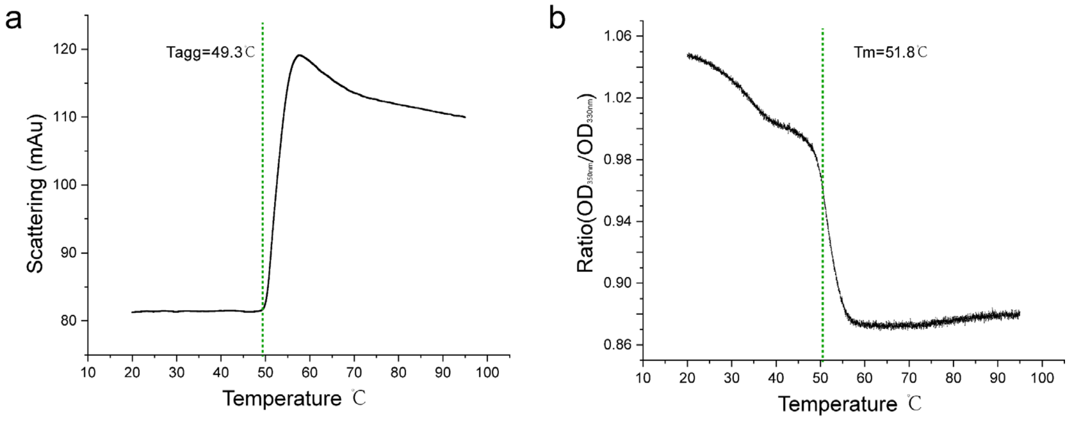

Supplementary figure 6

ESI-MS analysis results of macrolide antibiotics that cannot be hydrolyzed by EstX. They include 14-membered erythromycin, roxithromycin, clarithromycin, 15-membered azithromycin, tulathromycin, and 16-membered spiramycin, acetylisamycin, josamycin, midecamycin.

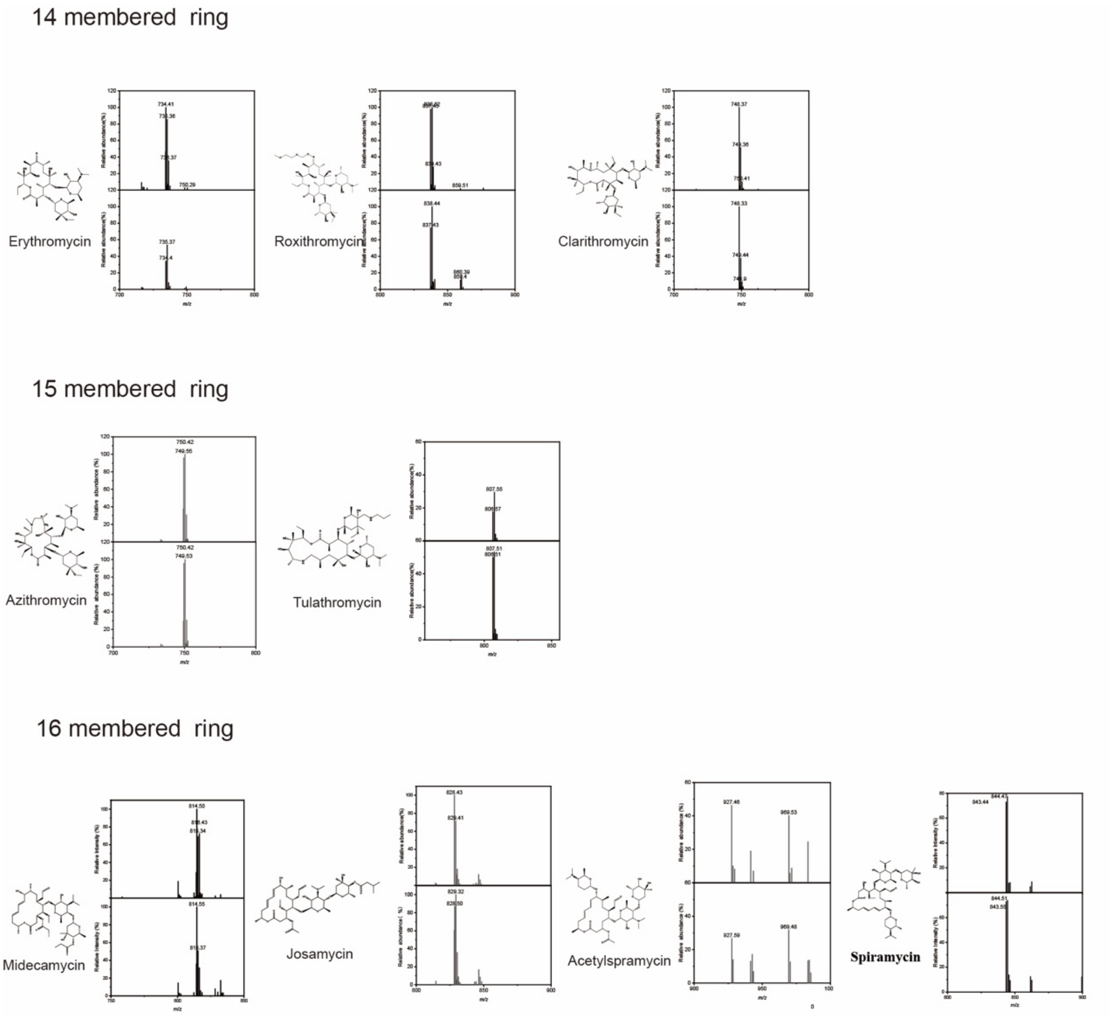

Supplementary figure 7

3D structure model of EstX, which was predicted by ColabFold. The different colors in the figure represent the confidence level of the prediction, red represents very high(>90), yellow represents confident (80), green represents ok(70), sky blue represents low(60), and blue represents very low (<50). It can be seen from the figure that most of the structure of EstX has a high confidence level, and only a few parts have a low confidence level. The structure of EstX contains multiple α-helices and β-sheets, as well as some irregular loops and chains

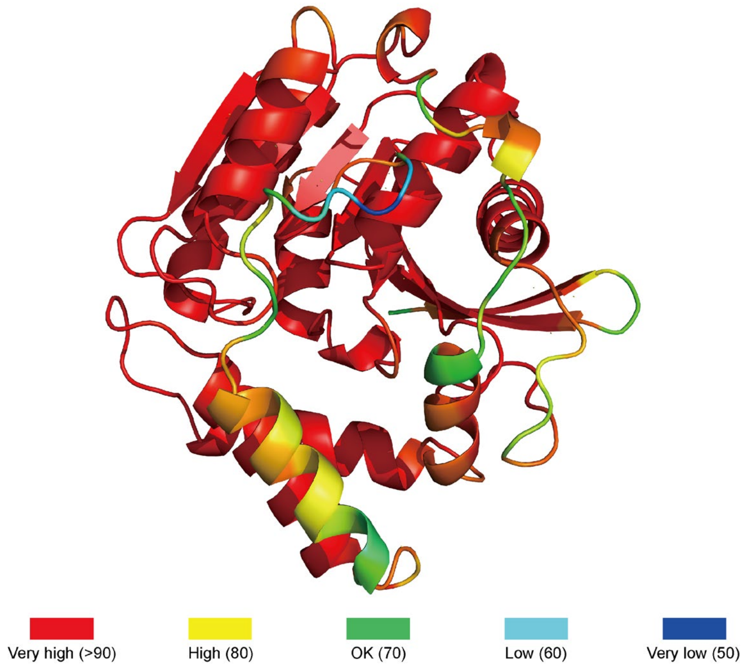

Supplementary figure 8

2D structure of the binding pocket of ligand (Tildipirosin、Tilmicosin and Leucomycin A5) and EstX, analyzed by Discovery studio software, showing different types of interactions

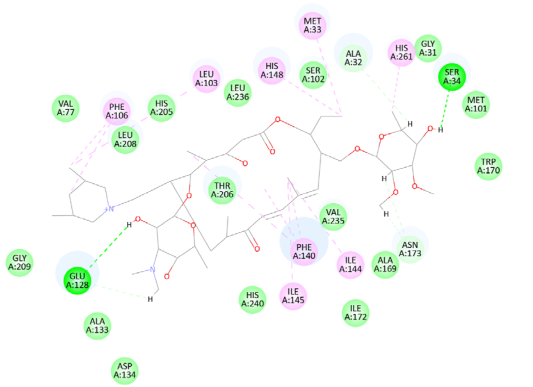

Tilmicosin

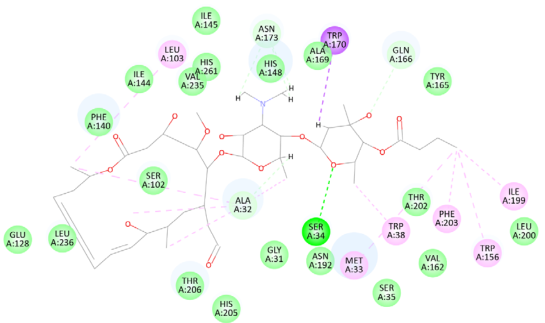

Leucomycin A5

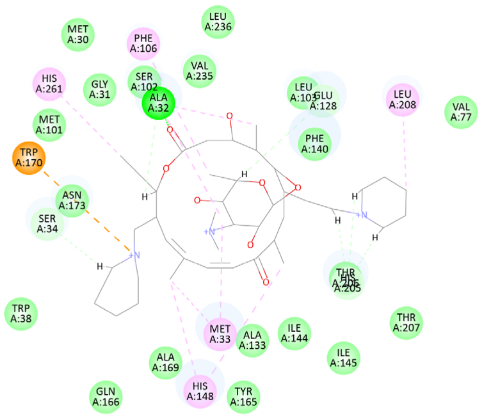

Interactions

- Conventional Hydrogen Bond
- Carbon Hydrogen Bond
- Van der Waals
- Pi-sigma
- Pi-Cation
- Alkyl
- Pi-Alkyl

Tildipirosin

Supplementary figure 9

Distance between macrolide ester bonds and catalytic amino acid in molecular docking results. Ligands include tylosin, tildipirosin, tilmicosin and Leucomycin A5.

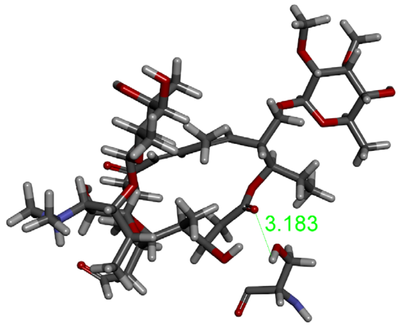

Tylosin

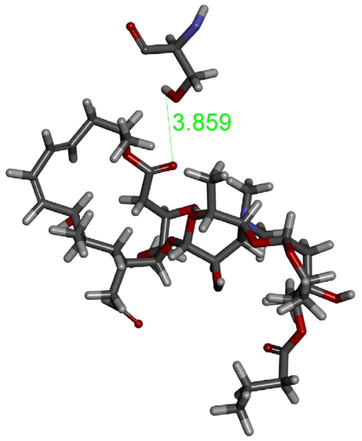

Leucmycin A5

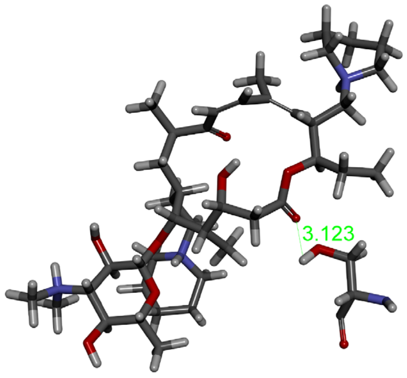

Tildipirosin

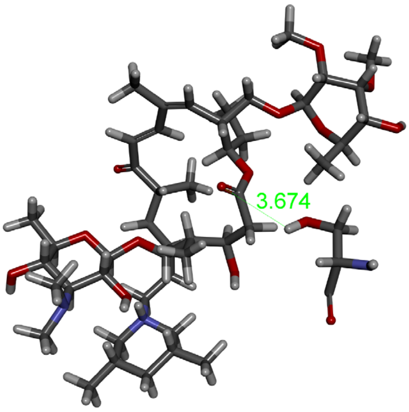

Timicosin

Supplementary figure 10

SDS-PAGE analysis showing the purified EstX mutations, lanes are labeled for purified S102A (1), D233A(2) and H261A (3) after IMAC purification.

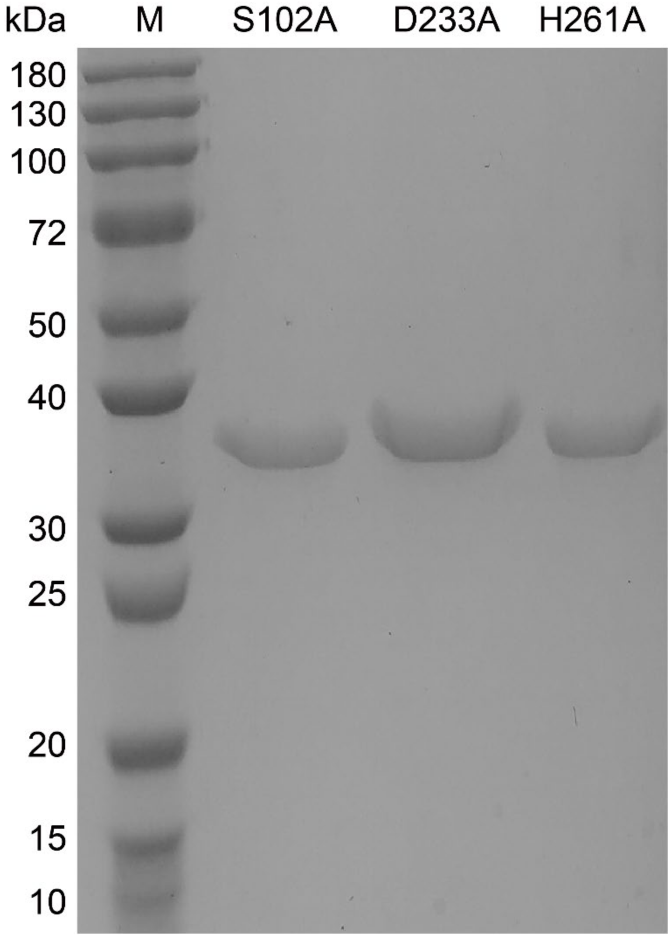

Supplementary table 1

Protein sequence shar sequence similarity with EstX in NCBI SwissProt database

Accession	Per. Ident(%)	Query Cover	Acc. Len	Description
Q54528.1	36.84	93%	298	Aclacinomycin methylesterase RdmC
Q55217.1	32.86	93%	298	Rhodomycin D methylesterase DauPl; 10-carbomethoxy-13-deoxycarminomycin ester
Q54809.1	32.40	93%	298	Rhodomycin D methylesterase DnrP; AltName: 10-carbomethoxy-13-deoxycarminomycin esteras

Note：Accession: The accession number of the protein in the NCBI SwissProt database, Ident(%): The sequence similarity percentage of the protein with EstX, Query Cover: The percentage of the EstX sequence covered by the protein sequence, Acc. Len: The length of the protein sequence (number of amino acids), Description: A brief description of the function or origin of the protein.

Supplementary table 2

Minimum inhibitory concentration (MIC) analysis of other class of antibiotics for *Escherichia coli* carrying either pET28a or pET28a-EstX. MIC values were determined using broth microdilution method.

classes	antibiotics(μg/mL)	*E.coli* pET28a	*E.coli* pET28a-EstX
quinolones	ciprofloxacin	＜0.5	＜0.5
	levofloxacin	＜0.5	＜0.5
β-lactams	cefotaxime	＜0.5	＜0.5
	amoxicillin	1	1
	cefoxifin	1	1
tetracyclines	terramycin	＜0.5	＜0.5
	doxycycline	＜0.5	＜0.5
lincosamides	clindamycin	＜0.5	＜0.5
	lincomycin	64	64
aminoglycosides	kanamycin	2	2
chloramphenicols	florfenicol	1	1

Supplementary table 3

Interactions of key amino acids between EstX and different ligands. Molecular docking was performed using Libdock and visualized in Discovery studio.

Interactions	Tildipirosin	Tilmicosin	Leucomycin A5	Tylosin
van der waals	Trp38,Asn173,Met101,Gly31,Met30,Ser102,Val235,Leu236,Leu103,Phe140,Val77,Thr207,Ile145,Ile144,Ala133,Tyr165,Ala169,Gln166	Val77,Leu208,His205,Leu236,Ser102,Gly31,Met101,Trp170,Ala169,Val235,Ile172,His240,Thr206,Asp134,Ala133,Gly209	Glu128,Leu236,Phe140,Ser102,Ile144,Val235,His261,Ile145,His148,Ala169,Typ165,Leu200,Thr202,Val162,Ser35,Asn192,Gly31,His205,Thr206	Ala139,Ile145,Leu236,Ser102,Met101,Gly31,Val235,Ser34,Trp38,Ala169,Gln166,His148,Trp156,Met33,Tyr165,Phe203,His205,Val77,Phe106
Pi-Cation	Trp170	-	-	-
Conventional Hydrogen Bond	Ala32	Ser34,Glu128	Ser34	Ser32
Carbon Hydrogen Bond	Ser34,Glu128, Thr206,His205	Ala32,Asn173	Ala32,Asn173,Gln166	Asn173,Thr206,Glu128
（Pi）Alkyl	His261,Phe106,Leu208,Met33,His148	Phe106,Leu103, His148,Met33,His261,Ile144,Ile145,Phe140	Leu103,Met33,Trp38,Phe203,Trp156,Ile199	Phe140,His261,Trp170,Leu208,Leu103
Pi-sigma	-	-	Trp170	-

Supplementary table 4

Predicted substrate binding pocket of EstX by prankweb.

name	rank	score	probability	residue_ids
pocket1	1	20.67	0.846	A_101 A_102 A_103 A_128 A_140 A_144 A_148 A_156 A_165 A_166 A_169 A_170 A_173 A_199 A_202 A_203 A_206 A_235 A_236 A_261 A_31 A_32 A_33 A_34 A_38
pocket2	2	1.75	0.031	A_102 A_103 A_106 A_128 A_205 A_208 A_210 A_32 A_77
