# Supplementary material for: The global distribution of the macrolide esterase EstX from the alpha/beta hydrolase superfamily
Source: Commun Biol. 2024 Jun 28;7:781. doi: 10.1038/s42003-024-06473-2 (PMC11214618; doi:10.1038/s42003-024-06473-2)
Supplement: Supplementary file 2 — Description of Additional Supplementary Files [file 42003_2024_6473_MOESM2_ESM.pdf]

Description of Additional Supplementary Files

File name: Supplementary Data

Description: The data that support the findings of this study.
